# Bacteraemia in sickle cell anaemia is associated with low haemoglobin: a report of 890 admissions to a tertiary hospital in Tanzania

**DOI:** 10.1111/bjh.13553

**Published:** 2015-06-17

**Authors:** Julie Makani, Josephine Mgaya, Emmanuel Balandya, Khadija Msami, Deogratias Soka, Sharon E. Cox, Albert N. Komba, Stella Rwezaula, Elineema Meda, David Muturi, Jesse Kitundu, Gregory Fegan, Fenella J. Kirkham, Charles R. Newton, Robert W. Snow, Brett Lowe

**Affiliations:** ^1^ Muhimbili University of Health and Allied SciencesDar‐es‐SalaamTanzania; ^2^ University of OxfordOxfordUK; ^3^ London School of Hygiene & Tropical MedicineLondonUK; ^4^ Muhimbili National HospitalDar‐es‐SalaamTanzania; ^5^ Kenya Medical Research Institute (KEMRI)‐Wellcome Collaborative ProgrammeKilifiKenya; ^6^ University College LondonLondonUK

**Keywords:** bacteraemia, sickle cell anaemia, haemoglobin, admission, Africa

## Abstract

Bacteraemia is a leading cause of morbidity in sickle cell anaemia (SCA), but information from studies in Africa is limited. We evaluated 890 admissions from 648 SCA patients at a tertiary hospital in Tanzania. Bacteraemia was present in 43 admissions (4·8%); isolates included *Staphylococcus aureus* (12/43; 28%), non‐Typhi *Salmonella* (9/43; 21%), *Streptococcus pneumoniae* (3/43; 7%) and *Salmonella* Typhi (2/43; 5%). Compared to SCA patients without bacteraemia, SCA patients with bacteraemia had significantly lower haemoglobin [71 g/l vs. 62 g/l, odds ratio 0·72 (95% confidence interval 0·56–0·91), *P* < 0·01]. Further exploration is needed of the relationship between anaemia and bacterial infections in SCA in Africa.

Individuals with sickle cell anaemia (SCA) are at an increased risk of invasive bacterial infections (Ramakrishnan *et al*, 2010). In the absence of preventions, bacterial infections are the leading cause of mortality in individuals with SCA, with the proportion of deaths from infection reported to be as high as 38% in the United States (Leikin *et al*, 1989) and 29% in Jamaica (Lee *et al*, 1995). Interventions with penicillin and vaccination against pneumococcal infections have successfully reduced mortality in these settings (Gaston *et al*, 1986; Knight‐Madden & Serjeant, 2001; Halasa *et al*, 2007). These interventions are not part of standard treatment and prevention for SCA in much of sub‐Saharan Africa, where the burden of SCA is highest. This is partly due to paucity of empirical data on the magnitude and pattern of bacteraemia in SCA (Obaro, 2009; Ramakrishnan *et al*, 2010). Nevertheless, studies from Kenya and Uganda have reported bacteraemia as a significant cause of morbidity in hospitalized SCA patients (Kizito *et al*, 2007; Williams *et al*, 2009). However, these studies are limited by the small number of SCA patients studied: 197 in Kenya (Williams *et al*, 2009) and 165 in Uganda (Kizito *et al*, 2007). In 2004, a SCA programme was established in Dar‐es‐Salaam, Tanzania, with prospective surveillance of one of the largest, single‐centre cohorts of SCA patients in Africa (Makani *et al*, 2011). Here, we present the prevalence and pattern of bacteraemia among 648 SCA patients who were admitted to a tertiary‐level hospital in an urban setting over a 3‐year period, between 1 January 2006 and 31 December 2008.

## Methods

### Study site and recruitment

The study was conducted at Muhimbili National Hospital (MNH) in Tanzania. This is the tertiary‐level, referral hospital for all individuals with SCA in Dar‐es‐Salaam. Standard care for SCA includes daily folic acid supplementation and treatment of malaria with artemisinin‐based combination therapy (for uncomplicated malaria) and quinine (for severe complicated malaria). Chloroquine was recommended, but was not offered to study subjects for malaria prophylaxis. Penicillin prophylaxis and pneumococcal vaccines were not the standard of care during the study period and were thus not offered to any study subject. The current study involved daily surveillance and enrolment of all SCA patients (known or suspected) who were admitted to the paediatric and adult medical wards during the study period. Enrolment was irrespective of clinical features at, and reason for admission. Ethical approval was given by Muhimbili University of Health and Allied Sciences (MUHAS; reference MU/RP/AEC/VOL XI/33) (Makani *et al*, 2011). Written informed consent was obtained from parents or guardians of children and from patients who were above 18 years of age.

### Laboratory methods

Sickle cell anaemia (SCA) was diagnosed using alkaline haemoglobin electrophoresis (Helena, Sunderland, UK) and high performance liquid chromatography (HPLC; Bio‐Rad, Hercules, CA, USA). In order to identify community‐ and not hospital‐ acquired bacteraemia, blood cultures were done within 24 h of hospitalization before antibiotics were given. Between 2 and 4 ml of venous blood for culture was collected from all study subjects by trained technicians using standard aseptic techniques, and cultures were performed using the BACTEC system (BACTEC; Becton Dickinson, Franklin Lakes, NJ, USA). The BACTEC bottles were weighed before and after inoculation for quality control. Positive blood cultures were sub‐cultured on standard media with the use of routine microbiological techniques. Positive quality control systems were done on the blood culture bottles as well as the media for sub‐culture to ensure growth of fastidious organisms. Once obtained, blood culture results were communicated to inpatient care physicians. Blood counts were performed with an automated cell counter (ABX Pentra 60, Horiba, Japan). Reticulocyte counts were performed using the new methylene blue method. Tests for liver and renal functions were done using an automated chemistry analyser (Roche Cobas Mira, New York, USA or Abbott Architect, New York, USA).

### Statistical methods

Data were analysed using STATA v10 (StataCorp, College Station, TX, USA). Each hospitalization was taken as a separate event, with some individuals being hospitalized more than once. The prevalence of bacteraemia was defined as the proportion of positive blood cultures in all blood cultures taken. 100 blood cultures (11·2%) grew non‐pathogenic organisms (contaminants) and were considered negative during analysis. These included coagulase‐negative *Staphylococcus aureus* (88), *Bacillus* species (11) and *Micrococcus* species (1). To determine factors associated with bacteraemia during hospitalization, the clinical and laboratory features of those with bacteraemia were compared to those without bacteraemia. In univariate analysis, multiple events with clustering of events within individuals were taken into account by applying a random effects model. *P* values of <0·05 were considered statistically significant.

## Results and discussion

Measuring the magnitude of bacterial infections in patients with SCA is critical for our efforts at reducing the morbidity and mortality in SCA. Here, we evaluated a total of 890 hospitalizations from 648 patients with SCA at a tertiary hospital in Tanzania (median age 8 years; range 1–43 years). The top three causes of admission in this cohort were pain, fever and anaemia. Bacteraemia was found in 4·8% of admissions. *Staphylococcus aureus* and non‐Typhi *Salmonella* were the most common isolates, although other bacteria, including *Streptococcus pneumonia* and *Salmonella* Typhi, were also isolated (Table 1). Compared to SCA patients without bacteraemia, presence of bacteraemia in SCA was significantly associated with lower haemoglobin [71 g/l vs. 62 g/l, odds ratio 0·72 (95% CI 0·56–0·91), *P* < 0·01]. SCA patients with bacteraemia were more likely to have symptoms of anaemia (easy fatiguability, palpitation, dizziness and headache), palpable liver and higher serum creatinine, although these associations did not reach statistical significance (Table 2).

**Table 1:** Bacterial isolates from blood of patients with sickle cell anaemia during 890 hospitalizations at Muhimbili National Hospital

Bacterial isolate	Isolates (*n*)	Percent (100× *n*/43)	Bacteraemia (100× *n*/890)
*Staphylococcus aureus*	12	27·9	1·3
Non‐Typhi *Salmonella*	9	20·9	1·0
*Streptococcus pneumonia*	3	7·0	0·3
*Streptococcus* species	3	7·0	0·3
*Escherichia coli*	3	7·0	0·3
*Klebsiella* species	3	7·0	0·3
*Pseudomonas* species	3	7·0	0·3
*Salmonella* Typhi	2	4·7	0·2
*Proteus* species	2	4·7	0·2
*Acinetobacter* species	1	2·3	0·1
*Aeromonas salmonicida*	1	2·3	0·1
*Morganella morganii*	1	2·3	0·1
Total number of isolates	43	100	4·8

**Table 2:** Clinical and laboratory features in individuals with sickle cell anaemia associated with bacteraemia during hospitalization

	No bacteraemia [*n* = 847/890 (95·2)]	Bacteraemia [*n* = 43/890 (4·8)]	OR (95% CI)	*P*
Age, years, GM ± SD	10·4 ± 8·4	10·4 ± 9·8	1·00 (0·96–1·04)	0·99
Symptoms				
Fever, *n*/*n* (%)	137/600 (22·8)	9/25 (36·0)	1·90 (0·78–4·63)	0·16
Pain, *n*/*n* (%)	187/478 (39·1)	11/23 (47·8)	1·43 (0·61–3·36)	0·42
Symptoms of anaemia,^a^ *n*/*n* (%)	50/600 (8·3)	5/25 (20·0)	2·75 (0·98–7·73)	0·06
Jaundice, *n*/*n* (%)	39/595 (6·6)	4/25 (16·0)	2·72 (0·90–8·17)	0·08
Examination				
Jaundice, *n*/*n* (%)	430/630 (68·3)	20/28 (71·4)	1·16 (0·49–2·73)	0·73
Pallor, *n*/*n* (%)	284/589 (48·2)	12/25 (48·0)	0·99 (0·46–2·13)	0·98
Temperature, GM ± SD	36·7 ± 0·7	36·9 ± 1·0	1·57 (0·94–2·62)	0·08
Febrile (>37·5°C), *n*/*n* (%)	64/627 (10·2)	4/25 (16·0)	1·68 (0·58–4·85)	0·33
SpO_2_, GM ± SD	97·7 ± 2·8	98·4 ± 2·2	1·12 (0·93–1·35)	0·22
Palpable spleen, *n*/*n* (%)	129/588 (21·9)	4/26 (15·4)	0·65 (0·22–1·94)	0·44
Palpable liver, *n*/*n* (%)	43/551 (7·8)	3/25 (12·0)	1·61 (0·45–5·72)	0·05
Laboratory features				
White blood cell count, ×10^9^/l, GM ± SD	18·5 ± 11·6	23·0 ± 15·9	1·02 (0·99–1·05)	0·06
Haemoglobin, g/l, GM ± SD	71 ± 16	62 ± 18	0·72 (0·56–0·91)	<0·01
Mean corpuscular volume, fl, GM ± SD	80·6 ± 9·9	82·1 ± 10·8	1·01 (0·97–1·06)	0·49
Red cell distribution width, %, GM ± SD	22·7 ± 4·3	22·6 ± 4·1	0·99 (0·90–1·09)	0·89
Platelet count, ×10^9^/l, GM ± SD	399·2 ± 206·1	371·1 ± 167·7	0·99 (0·99–1·00)	0·47
Reticulocyte count, %, GM ± SD	13·9 ± 6·6	13·9 ± 7·9	1·00 (0·93–1·07)	0·98
Bilirubin – Total, μmol/l, GM ± SD	67·6 ± 71·5	49·6 ± 34·1	0·99 (0·98–1·00)	0·23
Bilirubin – Unconjugated, μmol/l, GM ± SD	53·2 ± 41·3	30·4 ± 42·8	0·99 (0·98–1·00)	0·18
Aspartate transaminase, iu/l, GM ± SD	56·1 ± 41·3	56·2 ± 29·5	1·00 (0·99–1·01)	0·98
Alkaline phosphatase, iu/l, GM ± SD	265·7 ± 144·3	225·9 ± 119·6	0·99 (0·99–1·00)	0·28
Creatinine, μmol/l, GM ± SD	42·4 ± 26·9	58·4 ± 51·8	1·01 (1·00–1·02)	0·05
Lactate dehydrogenase, iu/l, GM ± SD	1068·2 ± 610·9	1141·4 ± 471·1	1·00 (0·99–1·00)	0·55
Haemoglobin F, %, GM ± SD	6·8 ± 5·2	7·9 ± 6·4	1·04 (0·96–1·12)	0·33

GM, geometric mean; SD, standard deviation; SpO_2_, peripheral oxygen saturation; OR, odds ratio; CI, confidence interval.

a Symptoms of anaemia included easy fatiguability, palpitation, dizziness and headache.

To our knowledge, our study is the first large‐scale, hospital‐based evaluation of bacteraemia in SCA in sub‐Saharan Africa (Ramakrishnan *et al*, 2010). Blood cultures were taken for all study subjects (known or suspected to have SCA) regardless of the presence of symptoms and signs suggestive of infection. This was justified by the high risk of bacteraemia among SCA patients reported in other studies (Zarkowsky *et al*, 1986; Wierenga *et al*, 2001; Williams *et al*, 2009; Ramakrishnan *et al*, 2010) as well as the tertiary‐level hospital study setting. The prevalence of bacteraemia reported in this study is similar to that observed in other SCA populations, including 6·6% in Kenya, 6·1% in Jamaica and 5·2% in the United States (Zarkowsky *et al*, 1986; Wierenga *et al*, 2001; Williams *et al*, 2009). The median age of the SCA population presented in this study was 8 years. This may account for the relatively infrequent isolation of *Streptococcus pneumoniae*, which is more prevalent among the younger SCA patients, predominantly those below 3 years of age (Zarkowsky *et al*, 1986).

In our study, SCA patients with bacteraemia were more likely to have symptoms of anaemia (easy fatiguability, palpitation, dizziness and headache) and had much lower haemoglobin levels during hospitalization. This is an important finding as anaemia was reported to be significantly associated with mortality in this setting (Makani *et al*, 2011). Further research is needed to explore the cause–effect relationship of anaemia and bacteraemia in this setting.

However, there are limitations to our study. First, the proportion of febrile patients and magnitude of bacteraemia reported in this study could be an underestimation because of potential prior usage of antipyretics and/or antibiotics at primary and secondary care facilities before referral to MNH. On the other hand, there may be a referral bias as patients with a known diagnosis of SCA may be more likely to seek care, leading to overestimation of bacteraemia. Second, the pattern of bacteraemia, particularly prevalence of *S. pneumoniae*, may have also been influenced by survival bias, as high‐risk children below 3 years of age may have died of pneumococcal bacteraemia before diagnosis of SCA or referral to the tertiary hospital. Further studies are needed to determine the prevalence and pattern of bacteraemia in SCA in different age groups and at primary and secondary health care facilities. Third, our surveillance of adult patients was limited to the medical, but not surgical wards, and thus may have missed adult SCA patients admitted to surgical wards with infective processes such as cellulitis and infected leg ulcers. Finally, the contamination rate of blood cultures in our study was high. 11·2% of blood cultures performed grew contaminants and were considered negative during analysis. Though similar to the rate of contamination reported in other studies (Kenyon *et al*, 2012), this may have lead to underestimation of the magnitude of bacteraemia in our cohort.

In summary, we have reported the prevalence of bacteraemia in hospitalized SCA patients from one of the largest studies of SCA in sub‐Saharan Africa. The prevalence as well as pattern of bacteraemia observed in our study is similar to that reported in other SCA populations. Presence of bacteraemia in SCA appears to be associated with lower haemoglobin. Further research is needed to explore the cause–effect relationship as well as the mechanisms of anaemia in SCA patients with bacterial infections. Furthermore, clinical trials are needed to determine locally appropriate interventions, particularly prevention (chemoprophylaxis and vaccination) as well as empirical antibiotics for treatment of individuals with SCA who are admitted to hospital with bacteraemia.

## Author contributions

JM designed the research, collected data, analysed the results and wrote the paper. EB assisted in writing of the manuscript. GF and FJK reviewed and analysed results and commented on draft manuscripts; CN, RWS and BL reviewed the results and commented on draft manuscripts. JM, DS, AK, SC, HM, JK, SR and EM collected data, reviewed the results and commented on draft manuscripts. DM managed the data and contributed to analysis. The authors have read and approved the final manuscript.

## Conflict‐of‐interest disclosure

The authors declare no competing financial or other interests.

## Funding

This work was supported by the Wellcome Trust, UK (JKM 072064; Project grant 080025; Strategic award 084538) and Kenya Medical Research Institute (KEMRI) – Wellcome Programme. RWS is supported by the Wellcome Trust as Principal Research Fellow (# 079080 and # 103602).
